# Effect of Pharmacogenetics Variations on Praziquantel Plasma Concentrations and Schistosomiasis Treatment Outcomes Among Infected School-Aged Children in Tanzania

**DOI:** 10.3389/fphar.2021.712084

**Published:** 2021-08-31

**Authors:** Rajabu Hussein Mnkugwe, Omary Minzi, Safari Kinung’hi, Appolinary Kamuhabwa, Eleni Aklillu

**Affiliations:** ^1^ Department of Clinical Pharmacology, School of Medicine, Muhimbili University of Health and Allied Sciences, Dar es Salaam, Tanzania; ^2^ Division of Clinical Pharmacology, Department of Laboratory Medicine, Karolinska University Hospital-Huddinge, Karolinska Institutet, Stockholm, Sweden; ^3^ Department of Clinical Pharmacy and Pharmacology, School of Pharmacy, Muhimbili University of Health and Allied Sciences, Dar es Salaam, Tanzania; ^4^ National Institute for Medical Research (NIMR), Mwanza Research Centre, Mwanza, Tanzania

**Keywords:** CYP2C19, schistosomiasis, treatment efficacy, adverse events, Tanzania, Praziquantel, CYP3A5, Africa

## Abstract

Studies on pharmacogenetics of praziquantel (PZQ) and its relevance on plasma drug concentrations and schistosomiasis treatment outcomes are lacking. We investigated the effect of pharmacogenetics variations of PZQ on plasma drug levels and schistosomiasis treatment outcomes among infected Tanzanian school-aged children. A total of 340 *Schistosoma mansoni* infected children were enrolled and treated with single-dose PZQ. Stool samples analysis was done by thick smear Kato-Katz technique, and treatment efficacy was assessed at 3-weeks post-treatment. Safety was assessed within 4 h after PZQ intake. Plasma samples were collected at 4 h post-dose, and PZQ and *trans*-4-OH-PZQ concentrations were quantified using UPLCMS/MS. Genotyping for CYP3A4*1B, *CYP3A5* (*3, *6, *7), *CYP2C19* (*2, *3, *17), and *CYP2C9* (*2, *3) were done by Real-Time PCR. The median age (range) of the study participants was 12 years (7–17). There was a significant association of CYP2C19 genotypes with PZQ concentrations and its metabolic ratio (*trans*-4-OH-PZQ/PZQ). PZQ concentration was significantly higher among *CYP2C19* (*2, *3) carriers than CYP2C19 *1/*1 and CYP2C19 *17 carriers (ultra-rapid metabolizers) (*p* = 0.04). The metabolic ratio was significantly higher among CYP2C19*17 carriers than *CYP2C19* (*2, *3) carriers (*p* = 0.01). No significant effect of *CYP3A4*, *CYP3A5*, *CYP2C19*, and *CYP2C9* genotypes on treatment efficacy or adverse events were observed. Baseline infection intensity and *CYP3A5* genotype were significant predictors of treatment associated-adverse events. In conclusion, *CYP2C19* genotype significantly affects plasma PZQ concentration and its metabolic ratio. For the first time, we report the importance of pharmacogenetic variation for the treatment of schistosomiasis, a neglected tropical disease.

## Introduction

Since 1984, praziquantel (PZQ) has been used in large-scale mass drug administration (MDA) programs for the treatment, control, and prevention of schistosomiasis worldwide (WHO, 2015). To date, PZQ is the only drug of choice recommended by the World Health Organization (WHO) (WHO, 2015). PZQ is reported to be safe and efficacious against all *Schistosoma* species, including *Schistosoma haematobium* (urogenital schistosomiasis) and *Schistosoma mansoni* (intestinal schistosomiasis). Globally, more than 800 million people are at risk of schistosomiasis infection, and about 250 million are infected and need treatment (Hotez et al., 2014; Mazigo, 2019). In Tanzania, schistosomiasis was first reported back in 1895 (Doumenge et al., 1984). To date, the disease is still endemic throughout the country despite ongoing interventions (Mazigo et al., 2012; Mnkugwe et al., 2020b). In 2017, approximately 99 million people of whom 81.1 million were school-aged children, received treatment worldwide (WHO, 2018). The WHO target is to control (heavy infections <5%) and eliminate (heavy infections <1%) the disease as a public health problem by the year 2025 (Gebreyesus et al., 2020).

As per WHO recommendation, preventive chemotherapy using mass PZQ treatment targeting school-going children is the main control strategy in endemic countries (WHO, 2015). PZQ MDA has played a significant role in reducing severe disease-associated morbidity and mortality in endemic settings (Andrade et al., 2017). However, the WHO recommended standard dose 40 mg/kg body weight of PZQ has been associated with varying results in both treatment efficacy, incidence, and profile of adverse events as reported in previous studies conducted in different populations (Kabuyaya et al., 2018; Mnkugwe et al., 2019; Mnkugwe et al., 2020a). In such studies, both high and low cure rates were reported, particularly with *Schistosoma mansoni* infection. The incidence and profile of treatment-associated adverse events also varied widely between populations. The causes for variability in drug response are multifactorial, including genetics, environment, and disease itself, which could potentially affect drug disposition (Wilkinson, 2005). Studies conducted in other infectious diseases such as malaria, tuberculosis, and HIV have reported variability in drug responses both treatment efficacy and adverse events in different populations due to genetic variations (Mugusi et al., 2012; Ngaimisi et al., 2013; Maganda et al., 2016).

However, studies to assess the contribution of genetic variations on PZQ plasma concentration and schistosomiasis treatment outcomes are lacking (Zdesenko et al., 2020). Although MDA poses a challenge for implementing individualized treatment, knowledge on how genetic variations affect PZQ blood levels and treatment outcomes is vital for improving treatment outcomes (Mukonzo et al., 2014; Mutagonda et al., 2017). Indeed, the utility of pharmacogenetic data to improve treatment outcomes has recently been intensified in Africa (Dandara et al., 2019). Furthermore, genetic variations can partly explain some of the reported variability on PZQ exposure, cure rates, and the incidence and profile of adverse events, as suggested previously (Bustinduy et al., 2016).

Factors such as age, pre-treatment infection intensity, and anemia are reported to affect schistosomiasis treatment outcomes among treated children (Zwang et al., 2017; Mnkugwe et al., 2019). Pharmacogenetic variations can potentially affect plasma drug levels and hence treatment efficacy and adverse events (Maganda et al., 2016; Ahmed et al., 2019). PZQ undergoes extensive phase 1 metabolism by *CYP3A4*, *CYP3A5*, *CYP2C19*, and *CYP2C9* enzymes to produce several metabolites, including 4-OH-PZQ (*Trans-* and *cis-*), which is a major metabolite of PZQ in humans (Wang et al., 2014; Nleya et al., 2019). The *trans*-4-OH-PZQ metabolite has been reported to possess antischistosomal activity (Kovač et al., 2017). *CYP3A4*, *CYP3A5*, *CYP2C19*, and *CYP2C9* are genetically polymorphic, displaying inter-individual variability in enzyme activity. The inherited defective/variant alleles may increase or decrease CYP enzyme activity resulting in variability in plasma drug levels. In pharmacokinetics -pharmacodynamics (PK-PD) studies, plasma drug concentration has been used as a surrogate marker for drug concentration at the site of action in the tissues (Bustinduy et al., 2016). High plasma drug exposure may increase the risk of adverse events, and low drug exposure results in poor therapeutic efficacy (Yimer et al., 2012). Therefore, genetic variations in CYP enzymes relevant for PZQ biotransformation can affect both PZQ plasma exposure and treatment outcomes (efficacy and safety).

To the best of our knowledge, no study has investigated pharmacogenetics variations of PZQ and its relevance on plasma concentration and schistosomiasis treatment outcomes despite reported variability in drug levels, cure rates, incidence, and profile of adverse events between treated populations (Zwang et al., 2017; Kabuyaya et al., 2018). We report the first pharmacogenetics study of PZQ and its relevance on plasma drug concentrations, treatment efficacy defined by cure rates, and adverse events among *Schistosoma mansoni* infected children treated with single-dose PZQ in Tanzania.

## Materials and Methods

### Study Design and Population

This was a prospective pharmacogenetics-pharmacokinetics-pharmacodynamics study aimed at investigating the effect of pharmacogenetics variations on PZQ plasma concentration and schistosomiasis treatment outcomes among *Schistosoma mansoni* infected children. The study was conducted between February 2017 and January 2018. The study was conducted in Nyamikoma village, North-western Tanzania. The Nyamikoma village is a rural area endemic for intestinal schistosomiasis (Mnkugwe et al., 2020b). The area has received five rounds of PZQ MDA targeting school-aged children. A total of 340 *Schistosoma mansoni* infected children (aged 7–17 years) were enrolled in this study.

### Data Collection Methods

Socio-demographic characteristics such as age and sex were obtained through interviews and school registries and recorded in case record forms (CRFs). Clinical data, including pre-treatment and post-treatment infection status, treatment-associated adverse events, body weight, and height and haemoglobin concentration, were recorded in the CRFs and categorized according to the existing WHO guidelines.

### Haemoglobin Concentration and Undernutrition Assessment

Pre-treatment haemoglobin concentration was determined by the HemoCue Hb 201 + analyzer (HemoCue AB Angelholm, Sweden) using finger-prick blood. Presence of anaemia was defined by haemoglobin (Hb) concentration of <11.5 g/dl (WHO, 2011). Undernutrition such as stunting and wasting were assessed by converting the height for age and body mass index (BMI) for age values into height for age Z score (HAZ) and BMI for age Z score (BAZ) using the WHO Anthro plus software version 1.0.4 (WHO, 2009). All values less than two standard deviations for both HAZ and BAZ scores were considered abnormal and classified as stunting and wasting, respectively.

### Therapeutic Procedures, Follow-Up and Safety Monitoring

Treatment was given following the WHO guidelines and recommendations for assessing the efficacy of an antihelminthic drug against schistosomiasis (WHO, 2013). Following a pre-treatment meal, a standard dose 40 mg/kg body weight of PZQ (Praziquantel 600 mg/tablet, Batch BZ6043, S Kant Health Care Ltd., India) was administered to each infected child as a directly observed treatment (DOT) (Mnkugwe et al., 2019). A follow-up visit was done 3-weeks post-treatment as recommended by the WHO guideline (WHO, 2013). Treatment-associated adverse events were monitored within 4 h after drug intake.

### Blood Samples Collection for DNA Extraction and Pharmacokinetics Analysis

A 2 ml pre-treatment whole blood sample was collected in EDTA tube from 340 study participants for genomic DNA extraction and stored at −80°C freezer. Another 2 ml whole blood sample was collected 4 h post-drug administration from 287 study participants in heparinized tubes and immediately centrifuged at 1,000 rpm for 10 min to obtain plasma, which was then kept at −80°C freezer until analysis. Blood and plasma samples were shipped to Karolinska Institutet (Stockholm, Sweden) for laboratory analysis.

### Laboratory Analyses

#### Thick Smear Kato-Katz Technique for *Schistosoma mansoni* Detection

The details of methods for stool sample processing and microscopic examination were presented previously (Mnkugwe et al., 2019). All laboratory procedures were done according to the WHO guidelines (WHO, 1991). Briefly, two fresh stool samples were collected from each participating child on two consecutive days and analyzed by thick smear Kato-Katz method both at pre-treatment and follow-up visit. The slides were then double read under light microscopy by trained and experienced laboratory technicians, and egg counts were recorded (Mnkugwe et al., 2019).

### Quantification of PZQ and *trans*-4-OH-PZQ Plasma Concentrations

#### Chemicals and Reagents

Rac-PZQ, an eleven-fold rac-deuterated-PZQ (rac-PZQ-d11) [internal standard (IS) for PZQ], *trans*-4-OH-PZQ and a five-fold *trans*-4-OH-PZQ (*trans*-4-OH-PZQ -d5) [internal standard (IS) for *trans*-4-OH-PZQ] were purchased from Toronto Research Chemicals (Toronto, Ontario, Canada). Acetonitrile, methanol, and formic acid of mass spectrometry (MS) grade were purchased from Merck (Darmstadt, Germany). Ultra-pure MilliQ water was prepared using a Milli-Q water purification system (Merck Millipore, Massachusetts, United States). Blank plasma was kindly supplied by the blood bank of the Karolinska University Hospital Huddinge (Stockholm, Sweden).

### Analytical Method

The UPLC-MS/MS method for quantification of PZQ and *trans*-4-OH-PZQ was adapted from Astra Zeneca laboratories (Sweden) and was recently used by Nleya et al., 2019 (Nleya et al., 2019) with minor modifications. In brief, plasma calibration samples were freshly prepared by spiking blank plasma samples with rac- PZQ and *trans*-4-OH-PZQ and were included in each analytical run. Quality control samples were also prepared by spiking plasma blanks to obtain low, medium, and high concentrations for both PZQ and *trans*-4-OH-PZQ. The quantification range of the method was set to 3.9–2,500 ng/ml for PZQ and 31.2–50,000 ng/ml for *trans*-4-OH-PZQ.

For extraction of analytes of interest, 50 µL of plasma samples went through protein precipitation with 150 µL of internal standards solution (25 nM of rac-PZQ -d11 and 25 nM of *trans*-4-OH-PZQ -d5 in 50:50 mixture of acetonitrile: methanol) and the mixture was vortexed for 3 min followed by centrifugation for 20 min at 3,220 g at 4°C. Then, 75 µL of the supernatant was diluted with 75 µL MilliQ water and 5 µL was injected into the UPLC-MS/MS for analysis. The chromatographic system was using an Aqcuity UPLC®HSS T3 column [2.1 × 50 mm, 1.8 µm (Waters, Ireland)]. The mobile phase consisted of solvent A (0.1% formic acid and 2% acetonitrile in water) and solvent B (0.1% formic acid in acetonitrile) with a flow rate of 0.8 ml/min. The column temperature was maintained at 60°C.

The chromatographic run was 4.7 min, starting at 4% of solvent B with an increase to 70% of solvent B at 2.6 min. From 3.1 min, the column was washed with 96% of solvent B until 4.1 min, with two dips to 4% of solvent B in the middle to ensure efficient washing. Column re-equilibration was done from 4.2 to 4.7 min but was in effect longer when including the injection time. T*rans*-4-OH-PZQ eluted first at a retention time of 1.15 min, followed by PZQ at 1.89 min. PZQ was monitored by the transition m/z 313.2 > 203.1 and the IS rac- PZQ -d11by 324.2 > 204.1 and for *trans*-4-OH-PZQ by the transition m/z 313.2 > 203.1 and the IS t*rans*-4-OH-PZQ -d5 by 324.2 > 204.1. Because of the very high concentrations of *trans*-4-OH-PZQ in the samples, a detuned (sub-optimized) MS method was used by decreasing the collision energy setting for that transition. Quantification of PZQ and *trans*-4-OH-PZQ was done using Target Lynx software (Waters). The calibration curves were constructed by linear regression of the analyte/internal standard area ratios, with a quadratic curve fit and an applied weighing of 1/x. A minimum of 12 calibration points were used, and calibrators were injected at start and end of each analysis. Three quality control samples were injected at regular intervals throughout the analyses. The PZQ and *trans*-4-OH-PZQ concentrations were estimated based on the ratio of the analyte peak area to the internal standard area.

Accuracy and recovery of the method was measured from three quality control samples each, at low (QCL), mid (QCM), and high (QCH) levels. For PZQ, recovery was 105% for QCL, 87% for QCM, and 100% for QCH at 5, 8.7, and 1% RSD, respectively. For *trans*-4-OH-PZQ the recovery was 104, 109, and 97.11 for the three QC levels, and accuracy was 2.6, 2.7, and 1.9% RSD. The precision for PZQ was measured by injection of six replicates and was 6.7% RSD at LLOQ, and 4.1% RSD at QCH. For *trans*-4-OH-PZQ, the area precision was 6.4% RSD at LLOQ and 5.3 at QCH. The calibration curves for both PZQ and *trans*-4-OH-PZQ had a coefficient of determination (*r* ^2^) of >0.98. No carry-over was detected for the compounds analyzed. The analytical method was partially validated according to the European Medicines Agency Guideline on bioanalytical method validation (EMA, 2009).

### DNA Extraction and Genotyping for CYP3A4, CYP3A5, CYP2C19 and CYP2C9

Genomic DNA was extracted from the peripheral leucocytes using the QIAamp DNA Midi Kit (Qiagen GmbH, Germany) according to the manufacturer’s instructions. Genotyping for common variant alleles for *CYP3A4* (*1B), *CYP3A5* (*3, *6, *7), *CYP2C19* (*2, *3, *17), and *CYP2C9* (*2, *3), which are relevant for PZQ disposition were determined as described previously (Maganda et al., 2016). In brief, genotyping was performed using TaqMan^®^ drug metabolism genotyping assay reagents for allelic discrimination (Applied Biosystems Genotyping Assays) with the following ID numbers for each SNP: C__11711730_20 for CYP3A4*1B (−392A > G, rs2740574), C__26201809_30 for CYP3A5*3 (c.6986A4G, rs776746), C__30203950_10 for CYP3A5*6 (g.14690G4A,rs10264272), C__32287188_10 for CYP3A5*7 (g.27131_27132insT rs41303343), C__25986767_70 for CYP2C19*2 (rs4244285), C__2,7861809_10 for CYP2C19*3 (rs4986893), C__469857_10 for CYP2C19*17 (rs12248560), C__25625805_10 for CYP2C9*2 (rs1799853), and C__27104892_10 for CYP2C9*3 (rs1057910). Genotyping was done by 7500 Fast Real-Time PCR (Applied Biosystems, United States). The final volume for each reaction was 10 μL, consisting of 9 μL TaqMan fast advanced master mix (Applied Biosystems, Waltham, MA, United States) and 1 μL genomic DNA. The PCR profile consisted of an initial step at 60°C for 30 s, hold stage at 95°C for 10 min, and PCR stage for 40 cycles step 1 with 95°C for 15 min and step 2 with 60°C for 1 min and after reading stage with 60°C for 30 s.

### Study Outcomes

The primary study outcome was the effect of *CYP3A4*, *CYP3A5*, *CYP2C19* and *CYP2C9* genotypes on PZQ, *trans*-4-OH-PZQ concentrations and metabolic ratio (*trans*-4-OH-PZQ/PZQ). The secondary outcomes were the effect of *CYP3A4*, *CYP3A5*, *CYP2C19* and *CYP2C9* genotypes on treatment efficacy (cure rate and eggs count reduction) and adverse events. The cure rate was defined as the proportion of infected children who were eggs positive for *Schistosoma mansoni* infection at baseline and turned negative at 3 weeks post-treatment (Mnkugwe et al., 2019). Eggs count reduction was defined by the mean percent change in eggs count per Gram between baseline and at 3 weeks’ post-treatment. An adverse event was defined as any symptom reported by a child, which is temporally associated with PZQ intake, but not necessarily causally related (Zwang et al., 2017).

### Statistical Data Analyses

Data was entered into Microsoft Excel and analyzed using the Statistical Package for Social Sciences (SPSS) version 20 (SPSS, IBM Corp, Armonk, NY, United States). Descriptive statistics were used for the analysis of both socio-demographic and clinical data. Socio-demographic characteristics were summarized into a frequency Tables as proportions for categorical data and mean ± standard deviations (SD) or median (range or Interquartile range- IQR) depending on the normality distribution of the data. Descriptive statistics were also used to analyze the treatment efficacy (i.e., cure rates) and treatment-associated adverse events as proportions in different CYP enzyme genotypes. Chi-square test was used to compare the genotype and allele frequencies between the observed and expected according to the Hardy-Weinberg equilibrium. The CYP2C19 genotype was categorized as CYP2C19 *17 carriers (*17/*17 or *1/*17), wild type (*1/*1), and *CYP2C19* *2, *3 carriers (*1/*2 or *1/*3 or *2/*17 or *3/*17 or *2/*2 or *2/*3 and *3/*3)*.* The means of the log-transformed PZQ, *trans*-4-OH-PZQ and t*rans*-4-OH-PZQ/PZQ concentrations were antilogged to obtain geometric means. One-way ANOVA was used to compare the geometric means of the PZQ, *trans*-4-OH-PZQ and t*rans*-4-OH-PZQ/PZQ concentrations between different CYP450 genotypes. The Pearson’s Chi-square test or Fisher’s exact test depending on test appropriateness was used for assessing the association between cure rates, adverse events and CYP genotypes. A univariate followed by multivariate regression analysis were used to identify the predictors of cure rate at week 3 post-treatment, and treatment-associated adverse events. Variables with *p* < 0.2 from univariate analysis were included in the multivariate regression model. One-way ANOVA was used to compare the mean percent change in eggs count (egg reduction) between different CYP genotypes. A negative binomial regression model was used to assess the predictors of eggs reduction at 3 weeks’ post-treatment. A variable with *p*-value < 0.05 was considered as a significant predictor.

## Results

### Baseline Characteristics of the Study Participants

A total of 340 children were enrolled in this study. The median age (range) in years of the study population was 12 years (7–17). Females were 53.2% of the study participants. The median baseline eggs/gram of stool (IQR) was 222 epg (96–468). At enrolment, about 22.4% of the study participants had anaemia (Hb < 11.5 g/dl). The prevalence of undernutrition as defined by stunting and wasting were 34.1 and 10.0%, respectively (Table 1).

**TABLE 1:** Baseline characteristics of the studied population.

Variable		N (%)
Age (years)	Mean ± SD	11.8 ± 1.7
	≤12 years	235 (69.1)
	>12 years	105 (30.9)
Sex	Male	159 (46.8)
	Female	181 (53.2)
Baseline eggs/Gram of stool	Median (IQR)	222 (96–468)
Baseline infection intensity	Light	87 (25.6)
	Moderate	152 (44.7)
	Heavy	101 (29.7)
Weight (kg)	Median (IQR)	30.2 (26.3–34.8)
Height (cm)	Median (IQR)	138.5 (130.4–144.0)
Stunting status (HAZ)	Stunted	116 (34.1)
	Not stunted	224 (65.9)
Wasting status (BAZ)	Wasted	34 (10.0)
	Not wasted	306 (90.0)
Haemoglobin concentration	Median (IQR)	12.7 (11.6–13.5)
Anaemia status	Anaemic	76 (22.4)
	Not anaemic	264 (77.6)

SD-Standard deviation; IQR-Interquartile range: BAZ-Body Mass Index (BMI) for Age Z score; HAZ: Height for Age Z score

### Genotypes and Alleles Frequencies

The overall genotype and allele frequencies for CYP3A4*1B, *CYP3A5* (*3, *6, *7), *CYP2C19* (*2, *3, *17) and *CYP2C9* (*2, *3) among Tanzanian children are summarized in Table 2. There were no significant differences in the observed and expected genotypes frequencies according to the Hardy Weinberg Equilibrium. CYP3A4 *1B allele occur at a highest frequency (66.7%), followed by CYP3A5*6 at 24.4%, and the lowest allele frequency was 0.4% for CYP2C9*2 (Table 2). Our previous *CYP3A* haplotype analysis in various black African population including Tanzanians indicated no linkage disequilibrium between the genotyped SNPs (Gebeyehu et al., 2011; Ngaimisi et al., 2014; Mutagonda et al., 2017). Likewise, there was no linkage disequilibrium between *2 and *3 alleles in *CYP2C9* and *CYP2C19* (Gebeyehu et al., 2011; Ahmed et al., 2019).

**TABLE 2:** Genotypes and allele frequencies for *CYP3A4*, *CYP3A5*, *CYP2C9* and *CYP2C19* in the study population.

Genotype		Frequency N (%)
CYP3A4*1B (-392A > G)	*1/*1	42 (12.3)
	*1/*1B	143 (42.1)
	*1B/*1B	155 (45.6)
CYP3A5*3 (c.6986A > G)	*1/*1	244 (71.8)
	*1/*3	84 (24.7)
	*3/*3	12 (3.5)
CYP3A5*6 (c.14690G > A)	*1/*1	192 (56.5)
	*1/*6	130 (38.2)
	*6/*6	18 (5.3)
CYP3A5*7 (27,131_27132insT)	*1/*1	279 (82.1)
	*1/*7	56 (16.4)
	*7/*7	5 (1.5)
CYP2C19*2	*1/*1	228 (67.1)
	*1/*2	103 (30.3)
	*2/*2	9 (2.6)
CYP2C19*3	*1/*1	328 (96.5)
	*1/*3	12 (3.5)
	*3/*3	0 (0.0)
CYP2C19*17	*1/*1	236 (69.4)
	*1/*17	92 (27.1)
	*17/*17	12 (3.5)
CYP2C9*2	*1/*1	337 (99.1)
	*1/*2	3 (0.9)
	*2/*2	0 (0.0)
CYP2C9*3	*1/*1	335 (98.5)
	*1/*3	5 (1.5)
	*3/*3	0 (0.0)
Allele	Minor allele	Percentage
CYP3A4*1B	*1B	66.7
CYP3A5*3	*3	15.9
CYP3A5*6	*6	24.4
CYP3A5*7	*7	9.7
CYP2C19*2	*2	17.8
CYP2C19*3	*3	1.7
CYP2C19*17	*17	17.1
CYP2C9*2	*2	0.4
CYP2C9*3	*3	0.7

The defective variant alleles occur at lower frequencies and the number of participants homozygous for defective variant alleles were very few in our study population. Therefore, to investigate impact of genotype on plasma PZQ metabolic ratio (Table 3) or treatment outcomes (Table 4), genotypes were categorized as normal metabolizers (*1/*1), and carriers of any defective variant alleles (intermediate or slow metabolizers) for *CYP3A4*, *CYP3A5* and *CYP2C9* genotype. For *CYP2C19*, participants were genotyped for both the high activity allele (2C19*17) and the loss of function alleles (2C19*2 and *3). Therefore, CYP2C19 genotype was categorized as CYP2C19*17 carriers (ultrarapid or rapid metabolizers i.e., *17/*17 or *1/*17), normal metabolizers (*1/*1), and carriers of *2 or *3 defective variant alleles (intermediate or slow metabolizers) as recommended by Clinical Pharmacogenetics Implementation Consortium (CPIC) Guidelines for CYP2C19 (Hicks, et al., 2017).

**TABLE 3:** Comparison of the geometric means of PZQ, *trans*-4-OH-PZQ concentrations (ng/mL) and metabolic ratio (t*rans*-4-OH-PZQ/PZQ) between CYP450 genotypes using One-way ANOVA.

Genotype		N	PZQ GM ± SD	p-value	Trans-4-OH-PZQ	p-value	trans-4-OH-PZQ/PZQ	p-value
*CYP3A4*	*1/*1	40	249.5 ± 3.3	0.88	9,299.7 ± 2.1	0.99	37.2 ± 3.0	0.86
	*1B carriers	247	258.2 ± 3.6		9,289.7 ± 1.9		36.0 ± 3.0	
*CYP3A5*	*1/*1	77	261.2 ± 3.5	0.89	9,462.4 ± 1.0	0.77	36.2 ± 2.8	1.00
	*3, *6, *7 carriers	210	255.3 ± 3.6		9,225.7 ± 1.9		36.1 ± 3.1	
*CYP2C19*	*17 carriers	79	191.9 ± 3.3	0.04	9,311.1 ± 1.8	0.92	48.5 ± 3.0	0.01
	*1/*1	109	267.9 ± 3.3		9,440.6 ± 1.9		35.2 ± 2.6	
	*2, *3 carriers	99	310.5 ± 4.0		9,099.1 ± 2.0		29.3 ± 3.3	
*CYP2C9*	*1/*1	279	258.2 ± 3.5	0.68	9,246.9 ± 1.9	0.37	35.7 ± 2.9	0.32
	*2, *3 carriers	8	214.3 ± 4.6		11,350.1 ± 1.6		52.9 ± 5.1	

GM–Geometric mean.

**TABLE 4:** Association of genotype with praziquantel efficacy (cure rates) and treatment-associated adverse events.

Genotype		Cure rates		*p* Value	Adverse events		*p* Value
		Cured N (%)	Not Cured N (%)		Yes N (%)	No N (%)	
*CYP3A4*	*1/*1	33 (12.0)	9 (14.1)	0.39	12 (13.2)	30 (12.0)	0.85
	*1B carriers	243 (88.0)	55 (85.9)		79 (86.8)	219 (88.0)	
*CYP3A5*	*1/*1	69 (25.0)	16 (25.0)	0.57	30 (33.0)	55 (22.1)	0.048
	*3, *6, or *7 carriers	207 (75.0)	48 (75.0)		61 (67.0)	194 (77.9)	
*CYP2C19*	*17 carriers	68 (24.6)	19 (29.7)	0.26	21 (23.1)	56 (26.5)	0.64
	*1/*1	104 (37.7)	28 (43.8)		39 (42.9)	93 (37.3)	
	*2, or *3 carriers	104 (37.7)	17 (26.6)		31 (34.1)	90 (36.1)	
*CYP2C9*	*1/*1	269 (97.5)	63 (98.4)	0.54	89 (97.8)	243 (97.6)	1.00
	*2, or *3 carriers	7 (2.5)	1 (1.6)		2 (2.2)	6 (2.4)	

### The Effect of CYP Genotypes on PZQ, *trans*-4-OH-PZQ Concentrations and Metabolic Ratio

The overall geometric means ± SD of PZQ, t*rans*-4-OH-PZQ and t*rans*-4-OH-PZQ/PZQ in the study population were 257.0 ± 3.6, 9,289.7 ± 1.9 and 36.1 ± 3.0 ng/mL, respectively. Comparison of the geometric means of PZQ, t*rans*-4-OH-PZQ and t*rans*-4-OH-PZQ/PZQ between different CYP450 genotypes are summarized in Table 3. There was a significant association between PZQ concentration, t*rans*-4-OH-PZQ/PZQ and *CYP2C19* genotype (*p* < 0.05). PZQ concentration was significantly higher among CYP2C19 *2, *3 carriers than wild type (CYP2C19 *1/*1) and CYP2C19 *17 carriers. The metabolic ratio (t*rans*-4-OH-PZQ/PZQ) was significantly higher among CYP2C19 *17 carriers than those who are CYP2C19 *1/*1 and CYP2C19 *2, *3 carriers. There was no significant effect of *CYP3A4*, *CYP3A5* and *CYP2C9* genotypes on PZQ, t*rans*-4-OH-PZQ concentrations and t*rans*-4-OH-PZQ/PZQ (*p* > 0.05) (Table 3).

### The Effect of CYP Genotypes on Treatment Efficacy

Overall, 81.2% (276/340) of the treated children were cured at 3-weeks post-treatment. There was no significant association between *CYP3A4*, *CYP3A5*, *CYP2C19*, and *CYP2C9* genotypes and cure rates (*p* > 0.05) (Table 4).

On multivariate logistic regression analysis, *CYP3A4*, *CYP3A5*, *CYP2C19* and *CYP2C9* genotypes were not significant predictors of cure at 3-weeks post-treatment. The model was a good fit with the Hosmer and Lemeshow test for the goodness of fit for multivariate analysis *χ* ^*2*^ = 6.40 and *p* = 0.60 (Table 5).

**TABLE 5:** Univariate and Multivariate logistic regression analysis for predictors of cure at 3 weeks’ post-treatment.

Variable		Cured N (%)	Univariate analysis		Multivariate analysis	
			cOR (95%)	*p*-value	aOR (95%)	*p*-value
Age (years)	≤12	190 (80.9)	1	0.82		
	>12	86 (81.9)	1.07 (0.59–1.94)			
Sex	Male	127 (79.9)	1	0.57		
	Female	149 (82.3)	1.17 (0.68–2.02)			
Baseline infection intensity	Light	72 (82.8)	1			
	Moderate	126 (82.9)	0.71 (0.34–1.46)	0.35		
	Heavy	78 (77.2)	0.70 (0.37–1.31)	0.27		
Anaemia	Yes	67 (88.2)	0.51 (0.24–1.09)	0.08	0.51 (0.24–1.09)	0.08
	No	209 (79.2)	1		1	
Stunting (HAZ)	Yes	96 (83.6)	0.78 (0.43–1.41)	0.41		
	No	179 (79.9)	1			
Wasting (BAZ)	Yes	30 (88.2)	0.55 (0.19–1.61)	0.27		
	No	246 (80.4)	1			
*CYP3A4*	*1/*1	33 (78.6)	1	0.65		
	*1B carriers	243 (81.5)	0.83 (0.38–1.83)			
*CYP3A5*	*1/*1	69 (25.0)	1			
	*3,*6,*7 carriers	207 (75.0)	1.00 (0.53–1.87)	1.00		
*CYP2C19*	*17 carriers	68 (24.6)	1		1	
	*1/*1	104 (37.7)	0.59 (0.28–1.21)	0.15	0.58 (0.28–1.21)	0.15
	*2,*3 carriers	104 (37.7)	0.96 (0.49–1.86)	0.91	0.97 (0.49–1.88)	0.92
*CYP2C9*	*1/*1	269 (97.5)	1	0.65		
	*2,*3 carriers	7 (2.5)	0.61 (0.17–5.05)			

cOR- Crude odd ratio; aOR–Adjusted odd ratio.

The overall mean percent change in eggs counts (egg reduction) at 3 weeks’ post-treatment was 101.6% ± 113.6 SD. There was no significant association between *CYP3A4*, *CYP3A5*, *CYP2C19* and *CYP2C9* genotypes and the mean percent reduction in eggs count at 3 weeks’ post-treatment (*p* > 0.05) (Supplementary Table 1). On negative binomial regression analysis, *CYP2C9*, *CYP2C19*, *CYP3A4* or *CYP3A5* genotypes were not significant predictors of mean percent reduction in eggs count at 3 weeks’ post-treatment (*p* > 0.05) (Supplementary Table 2).

### The Effect of CYP450 Genotypes on Treatment-Associated Adverse Events

In total, 26.8% (91/340) of the treated children experienced at least one treatment-associated adverse event within 4 h post-treatment. Abdominal pain (26.5%, 90/340) and vomiting (1.8%, 6/340) were the observed adverse event among the treated children. There was no significant association of *CYP2C9*, *CYP2C19*, or *CYP3A4*, or genotypes with treatment-associated adverse events as presented in Table 4. However, children carrying *CYP3A5* defective alleles (*3, *6, *7) had more incidence of adverse events than those who are wild type (CYP3A5 *1/*1) (*p* = 0.048) (Table 4 and Table 6)*.*

**TABLE 6:** Univariate and Multivariate logistic regression analysis for predictors of adverse events.

Variable		Adverse Events Yes N (%)	Univariate analysis		Multivariate analysis	
			cOR (95%)	*p*-value	aOR (95%)	*p*-value
Age (years)	≤12	68 (28.9)	1.45 (0.85–2.49)	0.18	1.59 (0.90–2.80)	0.11
	>12	23 (21.9)	1		1	
Sex	Male	38 (23.9)	1	0.26		
	Female	53 (29.3)	0.76 (0.47–1.23)			
Baseline infection intensity	Light	11 (12.6)	1		1	
	Moderate	40 (26.3)	0.22 (0.11–0.47)	≤0.001	0.20 (0.09–0.43)	≤0.001
	Heavy	40 (39.6)	0.55 (0.32–0.93)	0.03	0.50 (0.29–0.87)	0.01
Anaemia	Yes	25 (32.9)	1.47 (0.85–2.56)	0.17	1.43 (0.80–2.57)	0.23
	No	66 (25.0)	1		1	
Stunting (HAZ)	Yes	28 (24.1)	0.81 (0.49–1.36)	0.43		
	No	63 (28.1)	1			
Wasting (BAZ)	Yes	8 (23.5)	0.83 (0.36–1.89)	0.65		
	No	83 (27.1)	1			
*CYP3A4*	*1/*1	12 (28.6)	1			
	*1B carriers	79 (26.5)	0.90 (0.44–1.85)	0.78		
*CYP3A5*	*1/*1	30 (33.0)	1		1	
	*3,*6,*7 Carriers	61 (67.0)	0.58 (0.34–0.98)	0.04	0.62 (0.36–1.07)	0.09
*CYP2C19*	*17 carriers	21 (23.1)	1			
	*1/*1	39 (42.9)	1.08 (0.57–2.05)	0.81		
	*2,*3 carriers	31 (34.1)	1.32 (0.71–2.44)	0.38		
*CYP2C9*	*1/*1	89 (97.8)	1			
	*2,*3 carriers	2 (2.2)	0.91 (0.18–4.59)	0.91		

cOR- Crude odd ratio; aOR–Adjusted odd ratio.

On multivariate logistic regression analysis, *CYP3A4*, *CYP3A5*, *CYP2C19* and *CYP2C9* genotypes were not significant predictors of adverse events. Baseline infection intensity was the only significant predictor of treatment-associated adverse events (*p* < 0.05). Children with heavy infections had a significantly higher incidence of adverse events compared to those children with light and moderate infections. The model was a good fit with the Hosmer and Lemeshow test for the goodness of fit for multivariate analysis *χ* ^*2*^ = 4.43 and *p* = 0.73 (Table 6).

## Discussion

We investigated the effect of pharmacogenetics variations on PZQ pharmacokinetics and its treatment outcomes (efficacy and adverse events) among schistosomiasis infected school-aged children. The genotype and alleles frequencies of CYP3A4*1B, *CYP3A5* (*3, *6, *7), *CYP2C19* (*2, *3, *17), and *CYP2C9* (*2, *3) observed in this study were similar to what was reported previously in Tanzanian populations (Dandara et al., 2001; Mutagonda et al., 2017). Our key findings include 1) significant association of *CYP2C19* genotype with plasma PZQ concentrations and its metabolic ratio (*trans*-4-OH-PZQ/PZQ) and 2) no significant effect of *CYP3A4*, *CYP3A5*, *CYP2C19*, and *CYP2C9* genotypes on schistosomiasis treatment efficacy at 3-weeks post-treatment, 3) a borderline significant association of *CYP3A5* genotype with treatment-associated adverse events, being higher among carriers of defective variant alleles (*3, *6 and *7). Studies on the effect of CYP genotypes on plasma PZQ concentrations, metabolic ratio and schistosomiasis treatment outcomes are currently lacking (Zdesenko et al., 2020). To the best of our knowledge, this is the first study to investigate the effect of pharmacogenetics variations on plasma PZQ, *trans*-4-OH-PZQ concentrations and metabolic ratio (*trans*-4-OH-PZQ/PZQ) as well as treatment efficacy and safety.

PZQ, a racemic mixture of *R* and *S* enantiomers, is metabolized by CYP3A4, CYP3A5, CYP2C19, and CYP2C9 (Wang et al., 2014). We found a significant association of *CYP2C19* genotype with PZQ concentration; significantly higher plasma PZQ concentration among children carrying *CYP2C19* defective variant alleles than CYP2C19*1/*1 and CYP2C19 *17 carriers (ultra-rapid metabolizers). We also found a significant association between CYP2C19 genotype and metabolic ratio (*trans*-4-OH-PZQ/PZQ), where the metabolic ratio was higher among CYP2C19 *17 carriers than *CYP2C19* (*2, *3) carriers (Table 3). These findings may indicate that CYP2C19 but not CYP3A4, CYP3A5, or CYP2C9 is a major metabolic pathway for the formation of *trans*-4-OH-PZQ metabolite. Our results are in line with a previous *in vitro* study that reported CYP2C19 as a major metabolic pathway for the formation of 4-OH-PZQ metabolite (Li et al., 2003). A recent study by Nleya et al., reported CYP3A is responsible for the formation of X-OH-PZQ and not 4-OH-PZQ (Nleya et al., 2019), which further supports the findings of our study.

Our study found no significant effect of *CYP3A4*, *CYP3A5*, *CYP2C19*, and *CYP2C9* genotypes on schistosomiasis treatment efficacy (Table 4 and 5 and Supplementary Tables 1,2). CYP3A4 is a major metabolizing enzyme for most drugs used in tropical infectious diseases, including PZQ. In this study, CYP3A4 genotype was not significantly associated with schistosomiasis treatment efficacy. Although not statistically significant, higher cure rates among CYP3A4*1B carriers than CYP3A4*1/*1 genotype were observed (Tables 4 and 5). Likewise, although the association between *CYP3A4* genotype and PZQ concentrations was not statistically significant, those carrying *CYP3A4* defective alleles had high PZQ concentrations than those with wild type (CYP3A4*1/*1) (Table 3) and a high cure rate. In line with our observation, a low CYP3A4 enzyme activity has been reported previously in the Tanzanian population carrying *CYP3A4* defective alleles (Mirghani et al., 2006; Diczfalusy et al., 2008). Furthermore, a recent study conducted among the Tanzanian population reported a linkage disequilibrium (LD) between CYP3A4 *1B and CYP3A5 *1, which resulted in a low CYP3A4 enzyme activity (Mutagonda et al., 2017), which may explain the observed high cure rate in children carrying *CYP3A4* defective alleles.

*CYP3A5* is highly expressed among African populations than any other population, and its genotype determines the total CYP3A enzyme activity among black Africans (Gebeyehu et al., 2011; Ngaimisi et al., 2014). The *CYP3A5* defective alleles (*3, *5, *7) are associated with a low CYP3A enzyme activity in Tanzanian (Diczfalusy et al., 2008) and other African populations (Gebeyehu et al., 2011). In this study, *CYP3A5* genotype was not significantly associated with schistosomiasis treatment efficacy, although children carrying *CYP3A5* defective alleles (*3, *6, *7) were more cured than those with wild type genotype (CYP3A5 *1/*1) (Tables 4 and 5).

Despite a significant association between *CYP2C19* genotype and PZQ concentration and its metabolic ratio, *CYPC19* genotype was not significantly associated with schistosomiasis treatment efficacy among infected Tanzanian children following PZQ treatment. Although not statistically significant, children who carry *CYP2C19* defective alleles (*2, *3) were more cured than those who were CYP2C19 *17 carriers (ultra-rapid metabolizers) (Table 4). The observed genotypes and alleles frequencies of *CYP2C19* were similar to previous studies conducted among Tanzania populations (Dandara et al., 2001). Similarly, *CYP2C9* genotype was not significantly associated with schistosomiasis treatment efficacy in the study population. The frequencies of *CYP2C9* defective alleles (*2, *3) were found to be very low (<1%), similar to reports from other African populations (Bains, 2013). Since defective variant alleles of both *CYP2C9* and *CYP2C19* occur at a lower frequency in the black African population, larger sample size studies are needed to explore further the impact of genetic variation on schistosomiasis treatment outcome in the sub-Sharan Africa population.

Previous studies reported the importance of pharmacogenetic variations for treatment-associated adverse events among HIV and Tuberculosis infected (Mugusi et al., 2012; Ngaimisi et al., 2013; Yimer et al., 2014) or cancer patients (Ahmed et al., 2019) in Sub-Saharan Africa. Factors such as age, sex and pre-treatment infection intensity have been reported previously to affect schistosomiasis treatment outcomes (Zwang et al., 2017). In our study, baseline infection intensity and not *CYP3A4*, *CYP2C19* and *CYP2C9* genotypes was a significant predictor of adverse events following PZQ treatment. Baseline infection intensity was a significant predictor of adverse events following PZQ treatment similar to what was reported in previous studies (Erko et al., 2012; Mnkugwe et al., 2019). Heavily infected children experienced significantly more incidence of adverse events than children with light and moderate infections (Table 6). Unlike previous studies we found no significant association of anaemia or age with adverse events following PZQ treatment (Zwang et al., 2017; Mnkugwe et al., 2019). Interestingly in a univariate analysis, carriers of *CYP3A5* defective variant alleles had significantly higher adverse events (Tables 4 and 6). Children carrying *CYP3A5* defective alleles (*3, *6, *7) had more incidence of adverse events than those CYP3A5*1/*1 genotype (Table 4).

We recently reported significantly higher plasma exposure of S-PZQ than R-PZQ following treatment of PZQ in school children (Minzi et al., 2021). S-PZQ, the non-active component of PZQ, is the main contributor of the unpleasant taste of the drug causing nausea and vomiting in children (Meyer et al., 2009). We found no significant association of *CYP3A5* genotype with PZQ concentration or *trans*-4-OH-PZQ, the main metabolite of R-PZQ. Association of *CYP3A5* defective variant allele with a higher incidence of adverse events may indicate the importance of CYP3A for the metabolism of S-PZQ or other metabolites of R-PZQ not quantified in the present study, and hence our study limitation. Other CYP3A-dependent monohydroxy PZQ metabolites such as X-OH-PZQ reported recently (Nleya et al., 2019) may be responsible for the observed association of *CYP3A5* genotype with adverse events in our study. Future studies involving quantification of both R- and S-PZQ with their respective metabolites is needed to further explore the relevance of pharmacogenetic variation for treatment-associated adverse events.

## Conclusions

We report a significant association of *CYP2C19* genotype with plasma PZQ exposure and its metabolic ratio (*trans*-4-OH-PZQ/PZQ) in schistosomiasis infected children. Although no significant effect of *CYP3A4*, *CYP2C19* and *CYP2C9* genotypes was observed on schistosomiasis treatment efficacy and adverse events, the borderline association of *CYP3A5* genotype with treatment-associated adverse events requires further investigation. For the first time, our study highlights the importance of pharmacogenetic variation for pharmacokinetics and treatment outcomes of schistosomiasis, a neglected tropical disease affecting millions of children in sub-Sharan Africa.

## Data Availability

The original contributions presented in the study are included in the article/Supplementary Material, further inquiries can be directed to the corresponding author.
