# Supplementary material for: Effect of Pharmacogenetics Variations on Praziquantel Plasma Concentrations and Schistosomiasis Treatment Outcomes Among Infected School-Aged Children in Tanzania
Source: Front Pharmacol. 2021 Aug 31;12:712084. doi: 10.3389/fphar.2021.712084 (PMC8438567; doi:10.3389/fphar.2021.712084)
Supplement: Supplementary file 1 [file DataSheet1.docx]

Supplementary Material

# Supplementary Tables

**TABLE S1|** Comparison of the mean percent change in egg counts at 3 weeks’ post-treatment between different CYP450 genotypes.

| Genotype | | Mean % change in egg counts ±SD | p-value |
| --- | --- | --- | --- |
| *CYP3A4* | **1/*1* | 89.9±33.7 | 0.78 |
|  | **1B* carriers | 84.6±124.8 |  |
| *CY3A5* | **1/*1* | 92.8±27.0 | 0.49 |
|  | **3, *6, *7* carriers | 82.8±134.6 |  |
| *CYP2C19* | **17* carriers | 88.1±36.6 | 0.72 |
|  | **1/*1* | 89.8±31.3 |  |
|  | **2, *3* carriers | 78.3±191.9 |  |
| *CYP2C9* | **1/*1* | 85.1±118.7 | 0.84 |
|  | **2, *3* carriers | 93.6±18.0 |  |

**TABLE S2|** Negative binomial regression analysis for predictors of the mean percent reduction in eggs count at week 3 post-treatment.

| Parameter | Parameter estimates | | | | |
| --- | --- | --- | --- | --- | --- |
|  | B | 95% CI (B) | Std Error | Wald Chi-square | p-value |
| Constant | 4.87 | 2.08 - 7.67 | 1.43 | 11.67 | 0.001 |
| Age | 0.01 | -0.08 – 0.09 | 0.04 | 0.01 | 0.92 |
| Sex | 0.13 | -0.16 – 0.41 | 0.14 | 0.76 | 0.35 |
| Anaemia | 0.03 | -0.29 – 0.35 | 0.16 | 0.03 | 0.78 |
| Stunting | 0.07 | -0.23 – 0.36 | 0.15 | 0.19 | 0.63 |
| Wasting | 0.05 | -0.41 – 0.52 | 0.23 | 0.05 | 0.79 |
| *CYP2C9* | 0.05 | -0.72 – 0.83 | 0.39 | 0.02 | 0.88 |
| *CYP2C19* | -0.11 | -0.28 – 0.05 | 0.08 | 1.86 | 0.21 |
| *CYP3A4* | -0.04 | -0.42 – 0.34 | 0.19 | 0.04 | 0.85 |
| *CYP3A5* | -0.09 | -0.39 – 0.20 | 0.15 | 0.38 | 0.54 |
